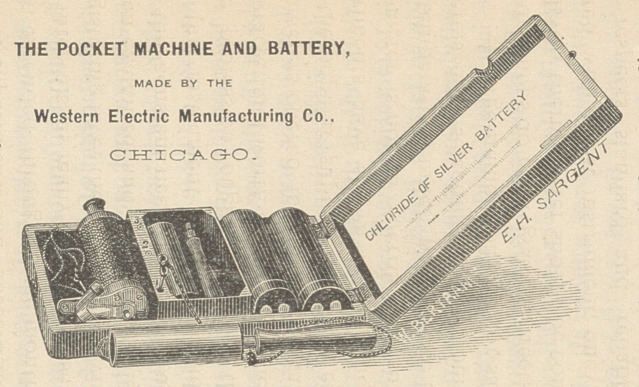# Transactions of the Chicago Society of Physicians and Surgeons, July 13 and 17

**Published:** 1874-08-15

**Authors:** Ralph E. Starkweather


### TRANSACTIONS OF THE CHICAGO SOCIETY OF PHYSICIANS AND SURGEONS.

### REGULAR MEETING, JULY 13, 1874.

*Reported by Ralph E. Starkweather, M.D.*

THE Society met, as usual, in the parlor of the Grand Pacific Hotel. Dr. John E. Owens, Vice President, in the chair. Dr. A. K. Norton was unanimously elected a member of the Society.

The paper read at the last meeting by Dr. P. S. Hayes, on Multilocular Sero-cystic Ovarian Tumor, an abstract of which appeared in the EXAMINER, showed that the operation by electro-puncture resulted in complete recovery.

Dr. Hyde reported a case of variola, modified either by antiseptic treatment or previous protection, of which the following is a brief summary:

The patient, a boy of five years of age, was seen on the second day of his illness, and had high fever; pulse 130; temperature 105 5-10 F.; constipation; coated tongue; sleeplessness and jactitation. The cheeks were suffused with a damask stain, with well-defined limits on every side; no redness of the fauces, sore throat or coryza. The boy had been vaccinated in early infancy, but upon inspection of the arm, a simple punctate cicatrix was visible, such as might have resulted from a wound of the arm by a shoemaker's awl.

On the fourth day, complete defervescence occurred. A general eruption then appeared, gradually extending, and invading the palms of the hands, the soles of the feet, and the pharynx. It rapidly passed through the stages of pimple and vesicle, until a well-defined umbilication occurred. The vesicles were generally discreet; the odor of small-pox was perceptible.

Some two years ago the attention of the Profession was attracted to a form of treatment by antiseptic solution, first reported in a medical periodical of Canada, and largely copied in our home journals. It consisted of one drachm of carbolic acid, ten of Squibb's pure medicinal sulphite of soda, and six fluid ounces of water. Dose for children, one-half to one drachm; for adults, a tablespoonful every three hours. Externally, a solution of two drachms of carbolic acid in three ounces of glycerine was employed. A febrifuge was also advised, of potassa chlorate, spirits of nitre and liquor ammoniæ.

The above treatment was modified, so that on the sixth day of this patient's disease, the following prescription was given:

℞.—Acidi carbolici cryst., ʒ i.

Sodæ sulphitis, (Squibb's medicinal.) ʒ x.

Aq. menth piperitæ, Aq. puræ, } aa. f ℥ iij.

M. Sig. One teaspoonful every three hours, day and night.

The external solution was made of less strength, and modified thus:

℞.—Acidi carbol., ʒ i.

Glycerin. f ℥ iv.

M. To be used on the exposed parts of the face and neck.

The result was as gratifying as surprising. Seventy-two hours afterwards, the ninth day of the disease, the little patient seemed practically cured of his malady. The eruption had everywhere subsided; no intumescence of the skin occurred between the vari; the itching was very slight, and the child was soon dressed and playing about the house. The subsequent history of the case is that of perfect restoration to health.

The speaker alluded to eight varieties of varioloid, given by Dr. Aitken, and contrasted the present case with them, developing several interesting points and deductions, and, in conclusion, said that there is as much evidence to show that the sulphite of soda is capable of controlling variola, as there is in favor of the remedial influence of the transfusion of blood. It should be carefully and faithfully tried in each case.

In remarking upon this use of the sulphite of soda, Dr. Chapman said that two years ago, at Ann Arbor, they had quite a siege of small-pox in the college, during which the patients were put under the treatment by the sulphites, with great success. Those treated so soon as the attack became apparent, were greatly benefited; the disease was modified in severity; there was no eruption on the palms of the hands, or the soles of the feet, though the patients had never been vaccinated. The sulphite of soda and the sulphite of magnesia were used: a drachm of either salt was placed in a glass of water, and the solution swallowed from time to time. A strong lotion of carbolic acid was used; the pitting was severe in two cases only.

Dr. F. H. Davis asked if carbolic acid was exhibited internally. Dr. Chapman said it was not, but a carbolic acid gargle was used in some of the worst cases.

Dr. F. H. Davis—I had supposed that the use of the carbolic acid internally had as much to do in contributing to successful results and relief as the sulphites. I have tried the sulphites in three or four cases, and none died, though two cases were of the confluent variola. The attack did not seem to be aborted; there was some pitting in the confluent cases; no carbolic acid was used externally.

By the President—It is to Dr. A. Fisher the Profession is indebted for establishing the dose of the sulphites. It is usually given in too small quantities at too infrequent intervals. Dr. Blake, in 1864, reported to the American Medical Association a case of pyemia, in a patient with pneumonia, pleurisy, and endocarditis with compound comminuted fracture of the tibia. He had ordered drachm-doses every four hours; at the end of thirty-six hours the patient had recovered from the pyemia; it was discovered, however, that four-drachm doses of the lime sulphate had been given instead of drachm doses. The medicine should be continued some time, to prevent relapses.

Dr. Fisher — I have frequently given one ounce of the soda sulphite in twenty-four hours—even an ounce and a half. The ordinary lime sulphite of the shops is totally unfit for use: that made by Dr. Squibb is the most suitable. In diarrhœa and cholera, a half-drachm of lime sulphite may be given every four hours. I have never seen bad effects from the use of the sulphites, and think I have aborted many cases which otherwise might have proved fatal. I give it in every disease of a septic nature. In one case there was an infant in whom vaccination was unsuccessful; I gave the sulphites, and no eruption followed. Sulphite of soda may be given in peppermint water, in an emulsion; it is not distasteful to children. The soda is preferable in cases of constipation. The sulphites are of no benefit in inflammatory diseases.

Dr. Andrews thought the sulphites were given too timidly. You must saturate the patient. Small doses are not so efficient as large.

Dr. Andrews addressed the Society in a partial review of the report of the Supervising Surgeon of the U. S. Marine Hospital Service, and compared this with the cases in the hospitals of London and Paris. The only subjects alluded to were those of the diseases of two races—the Teutonic and negro of this country, and the Teutonic (English) and Gallic (French) of Europe, and, also, that of their different physiognomy and facial angle and measurements.

His conclusions, in brief, were, that the British Teuton, as compared with the Gallic Parisian, is prone to alcoholic disease; the latter to venereal diseases. Some attribute the difference to influence of climate and food.

In this country, we have the two races side by side, and in the same relations as to climate and food, say for the past three hundred years they have grown side by side. In the marine hospital departments, the colored sailors, in large per centage, are under treatment for venereal diseases, while of the whites, much the larger per centage is treated for delirium tremens.

Dr. Hay—There is one view Dr. Andrews has omitted to take, which a close observation of the negro race for twenty years has led me to adopt. In the Teuton, the tendency to drink is partly hereditary; the negro in service, has been kept sober, regular and steady in habits, and, probably, his freedom from alcoholism is due to this exemption from hereditary alcoholism. I mean the transmission of certain modifications of the nervous system, due to the continued use of alcohol by successive generations.

Dr. Andrews—Did not doubt this, but had never been able to satisfy himself as to the degree of exemption.

The President read a deferred paper on a means of facilitating the introduction of Barnes' dilators. The devices of Skene and Bishop were referred to, and the method suggested was offered as additional to these.

Dr. Bartlett's plan consisted in the rolling up of the ordinary dilator about the supporting rod or tube, and the maintenance of that state of compactness till the introduction was effected, by means of a cord or tape adjusted about it, in such a manner as to permit of the ready release of the bag from the coils of the string.

The dilator having been exhausted of air by suction with the mouth, it is compactly rolled up on its long axis, and secured in this state by a temporary string at each end. A piece of wire, as a small stylet from a catheter, is then laid parallel with the bag and its supporting staff. The wire, bag and staff, are then lashed together by what Dr. Bartlett called a key-wire stitch. The cord is first tied around the wire at a point corresponding to the tube end of the dilator; it is then passed under and then over the bag and staff, and placed under the wire and looped over it, the direction of the string being thus reversed. The cord is then carried under and over the bag and staff, in a direction contrary to that of the first stitch, and is again carried under the wire and looped over it as before. This process of binding the bag on to the staff, by looping the string over the wire, is continued till the dilator is lashed to the staff in nearly its whole extent. The stitch is ended by taking several looped turns over the extremity of the wire; this latter is then withdrawn so that its end rests concealed just below the presenting extremity of the bag. The temporary strings are removed as the stitch is being made, or afterward. The dilator being introduced, the string is discharged from it by the withdrawal of the key-wire. Any form of cord will answer the purpose, but very narrow oiled tape is most suitable; the lashing should not be too tight. Dr. Bartlett thought it probable that this stitch might be usefully employed for other obstetrical or surgical purposes.

The meeting closed with the presentation of a testimonial by Dr. Hay, in behalf of many members of the Society, to the Secretary. It consisted of a set of Gouley's sounds, and a Nott's speculum, in recognition of that officer's long and faithful services.

The Society then adjourned.

---

REGULAR MEETING, JULY 27, 1874.

The Society met, as usual, in one of the parlors of the Grand Pacific Hotel, Dr. J. E. Owens, Vice President, in the chair. After requesting Dr. H. A. Johnson to take the chair, Dr. Owens read a paper on Thermometers, of which the following is a summary:

The experiments and observations had extended through three months, at intervals, as many as fifty-five self-registering fever thermometers having been examined and tested. In view of the constant necessity for the use of this instrument at the bed-side, the profession ought to know the character and worth of the fever thermometers offered for sale. Contrary to the popular supposition, these experiments have shown that the thermometer is not an unerring measure of temperature, unless proper care be exercised in selecting the thermometer. A large number offered for sale in the shops do not register correctly.

Every thermometer should be compared with a test instrument, one in all respects standard and reliable. The indices having been shaken to the same level, approximately, each lot was tested in warm water. In one lot of instruments examined, it was found that no two registered alike. In another lot, the errors were from three-fourths to one and one-fourth degrees Fahr. Each instrument remained beneath the tongue four minutes in this trial. In a fourth collection, the index of one thermometer could not be moved, while that of another had been lost in the mercury below and could not be disengaged, and no two gave the same registration, comparing them with a test-thermometer made by Beck, of London, of ascertained reliability and accuracy. In a sixth lot of eleven, only one registered correctly. The instruments above spoken of, were mostly of American manufacture, not marked by their maker's name, and almost entirely unreliable. They are unworthy the patronage of the profession.

Ten English thermometers, made by Maw, Son and Thompson, and Lynch & Co., when tested, varied only the one-fifth of a degree, and are more worthy of confidence. All instruments that are not known to be correct should be tested with a Casella thermometer. Mr. E. H. Sargent, of this city, a prominent dealer in instruments, has allowed the use of the English thermometers above spoken of.

Dr. Johnson—In regard to the index of a thermometer, I have long been satisfied that some thermometers will not always register the same in the same conditions of location and temperature. The mercury bubble adheres to the side of the tube, and does not flow readily; sometimes it goes up with a jump, and beyond its true level. There ought not to be a variation of even the one-half of a degree. Select an instrument in which the index will move readily, the more easily it does so the more reliable it will be. If the index move by starts—fitfully—it should be rejected.

Dr. Hyde, the Secretary, exhibited a cornu removed from the forehead of an Irish washer-woman, fifty-three years old, by Dr. McArthur. She had been operated upon by Dr. Andrews three years before, who removed, at that time, a cystic tumor from the anterior part of the left frontal bone. It was presumed that part of the cyst had been left *in situ*—"the root," as she termed it—since the growth returned where the cicatrix had formed. Six months prior to date, her son had sawn off a portion of the horn as large as that which was removed by the knife. The patient presented herself to the Dispensary, not because she suffered any pain, but because the growth was a source of annoyance to her, on account of the ridicule it excited among her neighbors. She has, at present, six or seven wens upon the occipital and parietal regions, which she refuses to have removed, for fear that similar horns would subsequently appear in the cicatrices that would result. The specimen presented was club-shaped at its extremity, and of even thickness.

As to the cause of the origin of these horns, Dr. Hyde said that Wilson and Fox regarded the cornu as a development from the sebaceous gland. Neuman, however, whose views of the pathology of this subject would be of very great weight and authority, says that the growth is due to the development from the papilla of the corium. It resembles a wart or a corn. The sebaceous follicle is an appendage of the hair. The hair, nail and tooth are identical, so far as the type is concerned—all proceed from a papilla. A French writer has reported one case in which the cornu had three extremities.

Dr. W. C. Lyman stated that he had removed a cornu an inch and one-quarter in length, from the centre of the lower lip.

Dr. Johnson exhibited a small Electro-Faradic machine with a chloride of silver battery, combined in a pocket-case, a cut of which is here shown. It weighs only fourteen ounces. This battery does not need to be prepared each time it is required for use; there is no acid or fluid of any kind, and it is much cleaner than others. Its strength is sufficient to run the helix of the largest size of the Kidder apparatus, and may be moderated at pleasure. It will run continuously ten, and even

THE POCKET MACHINE AND BATTERY,
MADE BY THE
Western Electric Manufacturing Co.,
CHICAGO.

twelve, hours, before needing renewal. The induced current is sufficient for medical purposes. There is, also, the extra current, and either form may be used; or, thirdly, the current resultant of the first two may be used. The battery itself is the chloride of silver and zinc. The elements are composed of a strip of zinc and another of *chloride* of silver. It is moistened by the chloride of sodium. These are enclosed in a hard rubber case, with a top screw cover, by which it is hermetically sealed. Two elements go with each case, and can readily and quickly be detached from the rubber case when exhausted, and new ones substituted. The machine is set in motion by moving an armature; metal handles, or electrodes are also provided.

Dr. N. S. Davis addressed the Society upon the subject of Diarrhœa in Children, substantially in the following words: He had treated the diseases of children for upwards of twenty years in this city, and said that the summer complaints of children had with pretty uniform certainty appeared upon the advent of the first week of consecutive and steadily hot, sultry weather. Such weather continuing some two or three days only, would not be enough to produce the general onset of the disease. This first week of hot weather may come as early as the last week in June, or not until the first, or even the second week in July. Occasionally, however, it will begin so late as the middle of July. Hardly any new cases originate after the last week in that month; there may be many cases from *relapse* in August, but few new cases. The incipient symptoms of almost all the cases can be traced to the first three weeks of hot weather. Dr. Davis in one instance kept a memorandum of cases occurring in his practice, where the first hot week ended June 25th. Within the next ten days of that date, he traced fifteen or more cases that dated the incipient diarrhœa directly into the last forty-eight hours of that hot week.

It is necessary to be careful in your inquiries, as often the milder at-attacks go unnoticed until a week has elapsed, especially among the laboring class. The mother will say that the child "didn't get bad till yesterday," though you will inquire and find that for the past fortnight it has been having from two to four thin stools daily.

I laid a plan before the proper Section of the American Medical Association, to obtain from the general government, in connection with the statistics and weather reports now furnished by the army signal service, reports as to the atmospheric electricity and ozone. A very competent and influential committee now has charge of this question. If we could secure physicians in every locality, who would be interested, laborious and faithful in their work, to keep watch and make a memorandum of the beginning of any initial symptoms of disease, stating the day, and, if practicable, the hour of beginning —tabulate them, and report the same to a common source, where again the reports might be condensed and generalized, we could then compare these statistics of disease with the atmospheric conditions obtained from the signal service, and thus see the exact relations between the two.

I have long been convinced that not otherwise could we get at the origin of endemic or epidemic diseases. These observations must not be delayed until the diseases come, but be made year after year, else we would be deceived. By comparing results for a series of years, we could arrive at some degree of certainty.

You must appreciate the actual pathological conditions which exist in any case before you can satisfactorily treat the same. I have long thought the essential conditions were extreme morbid excitability of the mucous membrane of the alimentary canal (perhaps likewise to some extent of the skin), coupled with a decrease of tonicity, or impairment of the vital affinity of atom for atom. As we increase temperature we separate atoms of matter; the human and living tissues are not an exception to this law of matter.

In diarrhœa it is seen in every degree, from the mere semi-fluid passage—three or four times a day—up to the rapid cholera morbus exudation, ejected by mouth and anus, so copiously that the patient dies exhausted in a few hours. The diarrhœa once started, the epithelium of the mucous membrane is disturbed and carried away; if only of moderate severity, there will be little points of abrasion or denuding; if the attack continues two or three weeks, these points take on more congestion; then we have inflammation, with more or less mucus in the discharges; there is some febrile reaction; skin hot; tongue dry, and a semi-dysenteric condition. Upon post-mortem examination, there will be found a grade of ileo-colitis. In some cases there is no fever, but the discharges and emaciation will continue, until, perhaps, in August, the patients become fatally exhausted and die.

Another class is one in which there is a more rapid change—the regular cholera infantum, with large loss of epithelium, as in cholera collapse. I have seen several cases terminate fatally in total collapse, in five hours—from a previous state of apparent health; one case in even three hours, but these are extreme cases. In such there will be little traces of disease found upon post-mortem examination; the intestine will look pale, rather than inflamed, and there may be a few ecchymoses. * * * *

Another class, and a rarer one, is not rapid in its onset or progress: the stools will be colored, turbid and watery—five or six in the twenty-four hours. The little patients melt down, though there is no heat or febrile reaction, gradually fail during two months, and die greatly emaciated. Is it an enteritis? A thorough search of the intestine will show it to be attenuated, translucent, with hardly a vestige of inflammation or congestion; there may be a few abrasions in the mucous membrane of the ilium and colon, and some of the mesenteric glands may be moderately enlarged and their centres somewhat softened.

Treatment.—Combine something directly soothing with an agent to give increased tonicity. An anodyne and tonic (not such a tonic as bark and iron), but a class that will increase contractility of the vascular system. For the past three or four years I have found nothing equal to carbolic acid, in cases of moderate or even violent vomiting in the early stages. The acid is never used alone, but the good effects are mainly due to it. It is not a specific, but forty-five out of fifty cases of vomiting will be checked by it, and it also moderates the diarrhœa. The following is the formula employed:

℞.—Acidi carbolici, cryst., gr. iv.

Glycerini, f ʒ ij.

Tr. opii camphoratæ, f ℥ j.

Aquæ camphoræ, f ℥ iii. M.

Dose.—Child, four to six months, ten drops hourly; if very sick, half-hourly. Child, six to twelve months, fifteen to twenty drops. Child, eighteen months, twenty-five drops, to be repeated every one or two hours until the stomach is quiet. The glycerine is put in to secure miscibility. Two fluid drachms of the aromatic spirits of ammonia may be put into the four-ounce mixture above given. Inquire as to the color of the stools: if light in color, turbid and watery, or gray and with no bile, you may order three or four of the following powders:

℞.—Hydrarg. chlor. mit., gr. ½, ad. i.
Pulv. opii, gr. 1-12.
Pulv. ipecac, gr. 1-6.
Sacchari alb. q. s. ft. chart. no. i.

Three of these, for the first day, will generally change the color of the stools. You will find but little urine in these patients, and calomel acts upon the general secretory apparatus. If the urine continues to be scanty, and you find the bile returning in the stools, omit the calomel, but bring in a diuretic, but not a purgative one. The spirits of nitrous ether was commended. If you overlook the urinary secretion when you check the diarrhœa, your patient may go into a stupor, with injected conjunctiva and sometimes convulsions. There will be no success unless you regulate most carefully the diet. For babies there can be nothing superior to their mother's milk, given frequently but in small quantities, so as not to fill up the stomach.

The most unpromising cases are those who are weaned or have no mothers. I have tried almost everything proposed, but have been forced back upon wheat flour and boiled milk in the form of a thin porridge. Do not dilute the milk; it has no other effect than to overload the stomach with more water. Never put in enough flour to make it thick when cold, and give a teaspoonful every half hour at first, and increase this quantity as the stomach becomes less sensitive.

Again, after the first stage of the attack is passed, in many cases the abdomen becomes hot; the palms of the hands dry; lips dry; there is occasional vomiting; eight or ten stools, in which there is more or less mucus of brown, reddish-brown or gray color, with here and there a streak or speck of blood. Then I go back to the turpentine emulsion, for which I have never found any substitute. The following is the formula:

℞.—Olei terebinthinæ f ʒ ij.

Olei gaultheriæ gtts. xv–xxx.

Tr. opii, f ʒ ij.

Pulv. accaciæ, } ʒ iv.

Sacchari alb., aa. }

Aquæ, q. s. ad. f ℥ iv.

M. Ft. emulsio.

The dose of the emulsion will be fifteen to thirty drops every three to six hours. Lengthen the interval between the doses in proportion to the interval between the stools: give every six hours; if no stool, in eighteen hours. If no stool in twenty-four hours, give no medicine until after a movement of the bowels occurs; then observe the nature of the stool, and if thin, repeat the medicine. If there should be no thin stools for several days or weeks, and then a relapse, resume the treatment with the emulsion.

In the last two years I have often used satisfactorily the zinc oxide (one-half to one grain for a child six to eighteen months of age), with minute doses of opium or Dover's powder; say the eighth to one-twelfth grain of powdered opium for a child one year old.

℞.—Zinci oxidi, gr. j.

Pulv. opii, gr. ⅛ to 1-12.

Hydrarg. chl. mitis, gr. ⅛.

M. Give the powder three times a day for two days, then omit the calomel and continue the other ingredients. In chronic cases—the patient weak, emaciated, urine scanty, with cerebral disturbance and bowels unchecked, I use the following:

℞.—Erigeron Canadensis, ℥ ss.
Morphiæ sulphatis, gr. j (anodyne).
Quiniæ tannatis, ℈j.
Aquæ bullientis, Oss.

M. Ft. infusio. Dose, one teaspoonful to a child of twelve to eighteen months of age, repeated every two, four or six hours.

Dr. Hyde—In diarrhœa we all look at the stools, and compare them with our idea of a normal stool. Will Dr. Davis tell us what such ought to be, in a child eight to twelve months, in summer, and while teething?

Dr. Davis—My idea is that a normal stool would be the same as it would be in a cold day. Teething has no influence upon it. I cannot understand how it is that the teeth become poisonous in hot weather, when in winter dentition goes on harmlessly. A natural stool varies from a soft semi-fluid, up to a well-formed consistent stool, retaining the shape of the intestine. If much beyond simple softness, it is abnormal. The color may be a little more yellow than usual. A great many slight cases of diarrhœa have stools of thinner consistency, unpleasant yellow color and very offensive odor.

As to the significance of the green stools, or their afterwards turning green, this may be due to the bile matter, but not always. The matter from the solitary glands of the large intestines may assume a green hue. You can test for bile in the stools.

Dr. Hyde had noticed that in classes of adults, those of different complexions had different color of stools. The same was true in regard to dogs: those white, had white colored stools; those black—the Newfoundland, for instance—had black colored stools. It is very much so in children. He laid less stress upon the color of stools than upon their homogeneity and consistence.

Dr. Davis—To prevent mistake, I will state that I look to the consistence and the homogeneity of the stools, and whether they are mucous. If the stools are consistent, I regard the patient as pretty nearly well. The color is nothing, unless very markedly changed, when it will indicate suppression of the secretions, which must not be neglected. The more you physic a patient the less likely you will be to have the true secretory organs collateral to the bowels acted upon, and the nearer you will approach to the true choleraic condition. Hence, if you want to act on secretions collateral to the bowels, keep the latter quiet, and increase excretory action by minute doses of alteratives and diuretics. Many a child has been killed by trying to carry off the secretions by active cathartic medication.

Dr. F. H. Davis had seen the statement that the diarrhœa of children was unknown on the continent of Europe, and was peculiar to American cities. Perhaps this suggests the origin of the disease. Some say it is malarial, and treat it with quinine in combination with opiates.

Dr. Simon—It seems to me that there is some coincidence in the relation between the color of the fœces and the complexion. Physiology teaches that the pigment cells take their origin in the liver.

Dr. Oleson was referred to as treating infantile diarrhœa by the potassium bromide, believing that it acted by allaying the irritability of the mucous lining of the bowel.

Dr. N. S. Davis—Some three years ago some reliable writer proposed a similar method of treatment. I have used it in a few cases, alone, and more often combined with paregoric in camphor water. The effects have been pleasant and favorable. In certain cases where he could not gain time enough to control the bowels, and there were cerebral symptoms, he had used the bromide and paregoric. There need be no fear in using the bromide alone, but there would probably be need of the paregoric in most cases.

Dr. H. A. Johnson had been in the habit, for the past eight years, of using the potassium bromide in cases where he did not desire to give opium to allay irritability, and in the early stage of diarrhœa. He knew of similar practice in the U. S. army.

The irritability is not a primary disease, but is the result of less tonicity. I'm quite sure the bromide will check the disease; it stimulates the urinary excretion, allays the nervous irritability and diminishes peristaltic action of the bowel. The potassium bromide may be given to a child one year of age, in two to four-grain doses, every four hours; and in doses pretty full, as compared with adults, often enough to procure rest.

Dr. Etheridge had had limited experience with the bromide, in the treatment of diarrhœa in children. He had found that the number of the discharges in the first twenty-four hours had not lessened, but that their character had improved and the child was better. If the use of the bromide was continued after the child was better, you would get brominism with its eruption.

Upon motion, the Society adjourned.

## Figures and Tables

**Figure f1:**